# The Impact of Universal Mask Use on SARS-COV-2 in Victoria, Australia on the Epidemic Trajectory of COVID-19

**DOI:** 10.3389/fpubh.2021.625499

**Published:** 2021-04-21

**Authors:** Valentina Costantino, Chandini Raina MacIntyre

**Affiliations:** ^1^The Biosecurity Program, The Kirby Institute, University of New South Wales, Kensington, NSW, Australia; ^2^College of Health Solutions, Arizona State University, Tempe, AZ, United States; ^3^College of Public Affairs and Community Solutions, Arizona State University, Tempe, AZ, United States

**Keywords:** Coronavirus – COVID-19, mask effectiveness, universal masks use, modeling study, outbreak control

## Abstract

**Objective(s):** To estimate the impact of universal community face mask use in Victoria, Australia along with other routine disease control measures in place.

**Methods:** A mathematical modeling study using an age structured deterministic model for Victoria, was simulated for 123 days between 1 June 2020 and 1 October 2020, incorporating lockdown, contact tracing, and case findings with and without mask use in varied scenarios. The model tested the impact of differing scenarios of the universal use of face masks in Victoria, by timing, varying mask effectiveness, and uptake.

**Results:** A six-week lockdown with standard control measures, but no masks, would have resulted in a large resurgence by September, following the lifting of restrictions. Mask use can substantially reduce the epidemic size, with a greater impact if at least 50% of people wear a mask which has an effectiveness of at least 40%. Early mask use averts more cases than mask usage that is only implemented closer to the peak. No mask use, with a 6-week lockdown, results in 67,636 cases and 120 deaths by 1 October 2020 if no further lockdowns are used. If mask use at 70% uptake commences on 23 July 2020, this is reduced to 7,961 cases and 42 deaths. We estimated community mask effectiveness to be 11%.

**Conclusion(s):** Lockdown and standard control measures may not have controlled the epidemic in Victoria. Mask use can substantially improve epidemic control if its uptake is higher than 50% and if moderately effective masks are used. Early mask use should be considered in other states if community transmission is present, as this has a greater effect than later mask wearing mandates.

## Significance of the Study

### The Known

Face masks were mandated in Victoria from 23 July 2020 onward, along with a 6-week stage three lockdown which commenced on 9 July 2020. Masks reduce the risk of infection with beta-coronaviruses.

### The New

Without masks, a 6-week lockdown and the current control measures would likely have resulted in a resurgence in Victoria by September 2020. Masks of modest to good quality with high enough usage (at least 50% of people) can substantially improve epidemic control. Early universal mask use results in a smaller epidemic than late mask use adoption.

### The Implications

The Victorian government's decision to mandate mask use is supported by our research. All efforts should be made to ensure the community have the information and means to obtain or make good quality cloth masks, along with instructions on correct mask use. High levels of mask use are required, and other states with community transmission should consider early adoption of universal facemasks. Masks were estimated to be 11% effective.

## Introduction

In late 2019, throughout most of 2020, and prior to the roll-out of vaccines, the available control measures for the COVID-19 pandemic were limited to non-pharmaceutical interventions (NPIs). NPIs are not enough on their own, and all interventions and measures must be used together to control a pandemic (1). Australia was one of the few countries in the world to achieve suppression of COVID-19, experiencing long periods without local transmission (2). Australia used border closures, travel bans (3), testing, contact tracing, quarantine, social distancing, and selected mask mandates.

The capital of Victoria, Melbourne, is the second largest city in Australia, with a population of 4.9 million. Melbourne experienced a resurgence of COVID-19 from June to August 2020 requiring a prolonged lockdown and a mask mandate. A mandate for masks in public spaces was issued close to the peak of the second wave on 23 July 2020.

A WHO-commissioned review (4) of NPIs against SARS, MERS-CoV, and SARS-CoV-2 showed a reduction in the risk of infection of 96% for N95 respirators and 67% for surgical or 12-layered cotton masks (4). However, the efficacy of different types of masks may vary widely. Respiratory protection can vary from respirators and medical masks to low quality cloth face coverings, with different materials and designs influencing their fit and filtering ability (5, 6). However, any mask can reduce exposure to aerosols in healthy people and emissions from infected people (7). Randomized clinical trial data for other viruses show protection using both mechanisms (8).

The control of COVID-19 has been challenging because of asymptomatic infection in up to 50% of all cases (9–12). Even in symptomatically infected people, 44% of transmission occurs in the 48 h prior to showing symptoms, and a further proportion on the first day of showing symptoms (13). Given that identifying potentially infectious people may be impossible in the community, universal mask use during periods of high transmission of SARS-CoV-2 may contribute to epidemic control. However, the role of mask use uptake, effectiveness, and the timing is unknown.

### Aims

To estimate the impact of universal community face mask use in Victoria, Australia, by mask use uptake, effectiveness, and timing.

## Methods

A mathematical model of SARS-CoV-2 transmission was developed for Victoria, with a population of 6.49 million people, with age distributed as per the Australian Bureau of Statistics (14). An age structured deterministic model was used, with compartments for people who are susceptible (non-immune); latent, not infectious; pre-symptomatic, infectious and diagnosed at two levels of infectiousness; pre-symptomatic, infectious and undiagnosed at two levels of infectiousness; symptomatic, undiagnosed; symptomatic, diagnosed; asymptomatic diagnosed and undiagnosed; quarantined contacts; isolated cases; hospitalized; admitted to ICU; and those who recovered or are deceased as a result of COVID-19. The varying levels of infectiousness was parameterized using longitudinal data on viral shedding, which show the peak of infectiousness in the 2 days prior to symptom onset and the first day of symptoms (13). Each of the compartments was divided into 16 age stratified groups each with a 5 year duration, ranging from 0 to 74 years old, plus an additional age group of 75+ years.

The model was simulated for 123 days starting on 1 June 2020 and lasted until 1 October 2020, and we started the epidemic with nine symptomatic cases reported on 1 June 2020 and two latent infected cases, which was chosen by fitting the modeled incidence of cases to the notification data (2). The rest of the Victorian population was considered susceptible. The force of infection is constructed to incorporate age-distribution and heterogeneity of contacts in different age-groups, so we used a contact matrix specific to Australia (15). We considered the average latent period to be 5.2 days (16), of which the last 2 days before symptom onset were considered to be infectious (13). In the baseline scenario, using published parameters for COVID-19, we assumed that 35% of cases were asymptomatic (17), and only 30% of them will be diagnosed when testing asymptomatic people. We assumed transmission could occur from people without symptoms, as this has been documented (18) and is consistent with credible estimates (17). We assumed a heterogeneous distribution of the infectiveness, with 44% of transmissions occurring in the last 2 days of the pre-symptomatic state (13), 36% on the first day of symptoms, and 20% distributed to the following 6 days of symptoms, with a total of 9 days of being in an infectious state.

Upon infection, a susceptible person (S) enters the latent, non-infectious compartment (E), and become infectious after 3.2 days and may be diagnosed (E^t^) or undiagnosed (E^u^). We assumed that traced contacts would be quarantined and that this would result in a 50% reduction in transmissions (19). When latent people become symptomatic, we assumed that the viral load is high the first day (I^1^,I^2^ for symptomatic previously untraced and traced; and A^1^ for people that never develop symptoms) and then decreases to lower infectious levels in the following 6 days (I^11^,I^22^, A^2^, respectively) (13). Symptomatic people will take 1 day to start isolation (Q) if they are identified early (such as through contact tracing or outbreak investigation) or 5 days (if they self-present without any active case finding by health authorities), respectively, (20), and in this state, once isolated, we assume no further transmissions. Depending on age-specific hospitalization and ICU required rates, people can be hospitalized (H) or use an ICU bed (ICU), before recovering (R) or before they die (D). We used the following differential equations to simulate the epidemic spread of COVID-19 in Victoria.

$$\begin{array}{l} \;dS_{i}/dt=\text{−}\left(1-m_{i}\right)*{\text{λ}}_{i}*S_{i}\\ \;dE_{i}/dt=\left(1-m_{i}\right)*{\text{λ}}_{i}*S_{i}-E_{i}/d_{0}\\ dE_{i}^{u}/dt=\left(1-\rho \right)*E_{i}/d_{0}-E_{i}^{u}/d_{1}\\ \;dE_{i}^{t}/dt=\rho *E_{i}/d_{0}-E_{i}^{t}/d_{1}\\ \;dI_{i}^{1}/dt={\left(1-g\right)*E}_{i}^{u}/d_{1}-I_{i}^{1}/d\\ \;dI_{i}^{2}/dt=\left(1-g\right)*E_{i}^{t}/d_{1}-I_{i}^{2}/d\\ dA_{i}^{1}/dt=g*\left(E_{i}^{u}/d_{1}+E_{i}^{t}/d_{1}\right)\;-A_{i}^{1}/d\\ dI_{i}^{11}/dt=I_{i}^{1}/d-\theta *I_{i}^{11}/d_{4}-\left(1-\theta \right)*I_{i}^{11}/d_{6}\\ dI_{i}^{22}/dt=I_{i}^{2}/d-I_{i}^{22}/d\\ dA_{i}^{2}/dt=\left(1-adr\right)*A_{i}^{1}/d-\;A_{i}^{2}/d_{6}\\ dQ_{i}/dt=adr*A_{i}^{1}/d+I_{i}^{22}/d+\theta *I_{i}^{11}/d_{4}\;-\left(1-h\right)\;\\ \;*Q_{i}/q_{2}-h*Q_{i}/d_{o1}\\ dH_{i}/dt=h*Q_{i}/d_{o1}-\left(1-icu\right)*H_{i}/dh\;-\;icu*H_{i}/d_{o1}\\ dICU_{i}/dt=\;icu*H_{i}/d_{o1}-ICU_{i}/d_{o1}\\ dR_{i}/dt=\left(1-{\mu 1}_{i}\right)*\left(1-\theta \right)*I_{i}^{11}/d_{6}+A_{i}^{2}/d_{6}+\left(1-{\mu 1}_{i}\right)\\ \;*\left(1-h\right)*Q_{i}/q_{2}+\left(1-{\mu 2}_{i}\right)*\left(1-icu\right)*H_{i}/dh\\ \;+\left(1-{\mu 3}_{i}\right)*ICU_{i}/d_{o1}\\ dD_{i}/dt={\mu 1}_{i}*\left(1-\theta \right)*I_{i}^{11}/d_{6}+{\mu 1}_{i}*\left(1-h\right)*Q_{i}/q_{2}\\ \;+{\mu 2}_{i}*\left(1-icu\right)*H_{i}/dh+{\mu 3}_{i}*ICU_{i}/d_{o1}\; \end{array}$$

The force of infection is described as

$$\begin{array}{l} \;{\text{λ}}_{i}=\sum_{j=1}^{18}\frac{\beta_{1}*c_{i,j}*E_{j}^{u}}{N}+\sum_{j=1}^{18}\frac{\beta_{2}*c_{i,j}*E_{j}^{t}}{N}\\ \;+\sum_{j=1}^{18}\frac{\beta_{3}*c_{i,j}*(I_{j}^{1}+I_{j}^{2}+A_{j}^{1})}{N}\\ \;=\sum_{j=1}^{18}\frac{\beta_{4}*c_{i,j}*(I_{j}^{11}+I_{j}^{22}+A_{j}^{2})}{N} \end{array}$$

Where $\beta_{1}=\frac{0.44\;*\;R0}{d_{1}}$ for latent undiagnosed contacts, $\beta_{2}=\frac{\beta_{1}}{2}$ for latent diagnosed and home quarantined (50% reduction in R0), β_3_ = 0.36 * *R*0 for the first day of symptoms and $\beta_{4}=\frac{0.2\;*\;R0}{d_{6}}$ for the following 6 days of symptoms. *c*_*i,j*_ is the age-specific contact matrix adapted from (15) for Australia, and *N* is the total population. We then added the mask use reduction in transmissions as a combination of the proportion wearing it and mask effectiveness to reduce the force of infection.

The contact matrix projected for Australia (15) shows increased values on the diagonal, therefore the highest contact rates are between people in the same age-group, followed by rates between the youngest children and adults. The lowest contact rates are in those aged over 75 years.

All values for the parameters used are listed in Table 1.

**Table 1:** Model parameters.

**Parameter**	**Symbol**	**Value**	**References**
Basic reproduction number	R0	2.5	(17)
Latent pre symptomatic period	*d*_0_ + *d*_1_	3.2 not infectious +2 infectious = 5.2	(16)
Infectious period	*d*_1_ + *d* + *d*_6_	2+1+6 = 9 days of which 2 presymptomatic, first day symptomatic with higher transmissions and following 6 days symptomatic with lower transmissions	(13, 17, 21–24)
Time to get isolated once symptomatic	*d* + *d*_4_	1+4 =5 days	(20)
Effectiveness of home quarantine	R0/2	50% reduction in the R0	(19)
Duration of isolation	*q*_2_	20 days	
Reduced transmission rates mask use as a combination of proportion wearing it by mask effectiveness	*m*_*i*_	70% wearing masks 67% effective is the base case scenario; we tested values from 20 to 90% for the proportion of the population wearing it and mask effectiveness	(4, 13, 25)
Proportion of asymptomatic or very mild infectious	*g*	35%	(9, 17, 26)
Asymptomatic diagnosed rate	*adr*	30%	
Proportion of contacts identified for home quarantine	ρ	80%	(27)
Proportion of symptomatic people that get isolated after 5 days	θ	90%	(27)
Age-specific case fatality rate (%) for the 16 age groupsFor severe hospitalized and ICU admitted people	μ1_i_ μ2_i_ = 2 * μ1_i_ μ3_i_ = 3 * μ1_i_	0, 0, 0.2, 0.2, 0.2, 0.2, 0.2, 0.2, 0.4, 0.4, 1.3, 1.3, 3.6, 3.6, 8, 14.8	(28)
Hospitalization rates	*h*	0–4 years old 0.003 5–19 years old 0.001 20–49 years old 0.025 50–64 years old 0.074 65–74 years old 0.122 75+ years old 0.165	(29)
ICU rates from hospitalization	*icu*	14.2% in the 54–79 years old and we used the age specific hospitalization rates to estimate the age distribution of the ICU rates, we get 0.0013 (0.13%) for 0–19 years old hospitalized 0.0337 (3.37%) for 20–49 and 0.142 (14.2%) for 50+	(30)
Time in ICU	*d*_*o*1_	5 days	
Time in hospital	*dh*	15 days	

We modeled the effect of the stage three lockdown which started on 9 July 2020, lasting for 6 weeks until 20 of August 2020 (31). This stipulated that people living in “*metropolitan Melbourne and the Mitchell Shire you must stay at home. You can only leave home for one of the four reasons—shopping for food and supplies, care and caregiving, exercise, and study and work, if you can't do it from home* (31).” We assumed a 50% reduction in mobility and contact as a result of this, given that not all of Victoria was in lockdown, and people in lockdown are allowed to go to work or to the shops. This is consistent with mobility data from the epidemic in New York City at a similar stage (32). The model assumes that 80% of all close contacts of diagnosed cases are identified through contact tracing and are quarantined, and 90% of symptomatic cases are isolated after 5 days (27). These are held as fixed in the model so that the only variation tested is the use of masks. It is necessary to consider these routine disease control interventions, or the size of the epidemic will be unrealistically large. In the base case scenario, we tested medical masks. The effectiveness of wearing medical masks on disease transmission is estimated from a meta-analysis of mask use against beta coronaviruses which found that wearing a medical mask or high-quality cloth mask is 67% effective in the community setting (4). We conducted a sensitivity analyses on the proportion of people wearing masks (20–90%) and mask effectiveness in the range of 20–90% to allow for a range of choices from poor quality cloth face coverings to N95 respirators (4). There are no data on cloth masks other than 67% for 12-layered cloth masks against SARS (4), and no efficacy for a 2-layered cloth mask (33). Given the variability of home-made cloth masks, from a scarf to custom-designed masks, we assume a range of effectiveness of 20–67%. This sensitivity analysis also addresses the uncertainty around the baseline case estimate of mask effectiveness. Finally, we used estimates of mask use of 75% in Victoria (34) and fitted the model to the number of cases observed by 1 October 2020 (35) to estimate the real-world effectiveness of masks during the second wave.

## Results

**Figure 1:**
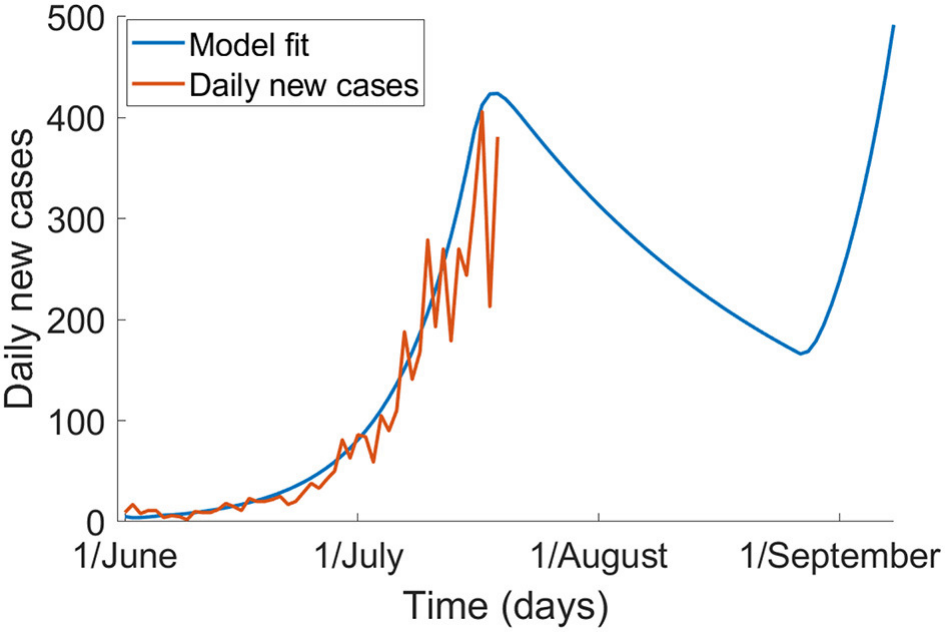
The epidemic of COVID-19 in Victoria without mask use, and with a 6-week lockdown from 9 July to 20 August (blue) and the observed epidemic up to 19 July 2020 based on notification data (red).

Figure 1 shows the epidemic without mask use, with the effect of a 6-week stage three lockdown from 9 July until 20 August. The model fits well to data observed from 1 June to 19 July. We show that the epidemic peaks around 19 July and decreases through early to mid-August but will increase again following lifting of the lockdown. With no further outbreak control measures implemented, by 1 October, there may be 67,636 cases and 120 deaths in total.

**Figure 2:**
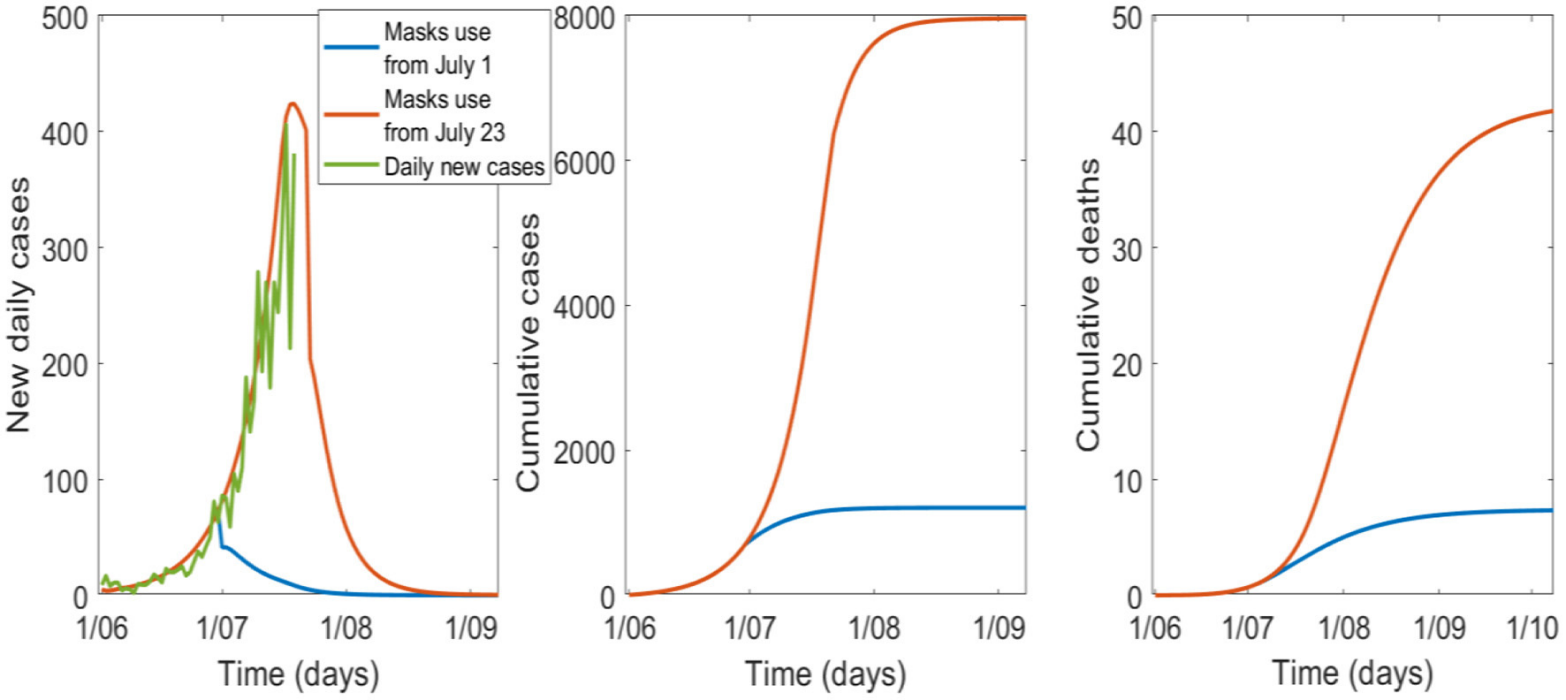
The effect of the timing of universal mask use with a 6-week lockdown and 70% uptake, starting on 1 July or 23 July in Victoria.

If universal masks use with a 70% uptake is added to the 6-week lockdown, the epidemic peaks around 19 July and can be controlled by September 2020 (Figure 2, Table 2). If universal masks use started on 1 July, compared to 23 July, it may have reduced cases and deaths from 7,961 to 1,209, and from 42 to 7, respectively, with a smaller peak in early July.

**Table 2:** Cases and deaths in the no-mask scenario compared to starting masks on 1 July or 23 July 2020.

**Start wearing masks**	**Cases by 1st of October**	**Deaths by the 1st of October**	**Reduction in cases and deaths (%)**
No mask and 6-week lockdown	67,636	120	Base value
1 July, 70% wearing a mask	1,209	7	−98.2% in cases −94.2% in deaths
23 July, 70% wearing a mask	7,961	42	−88.2% in cases −65% in deaths

**Figure 3:**
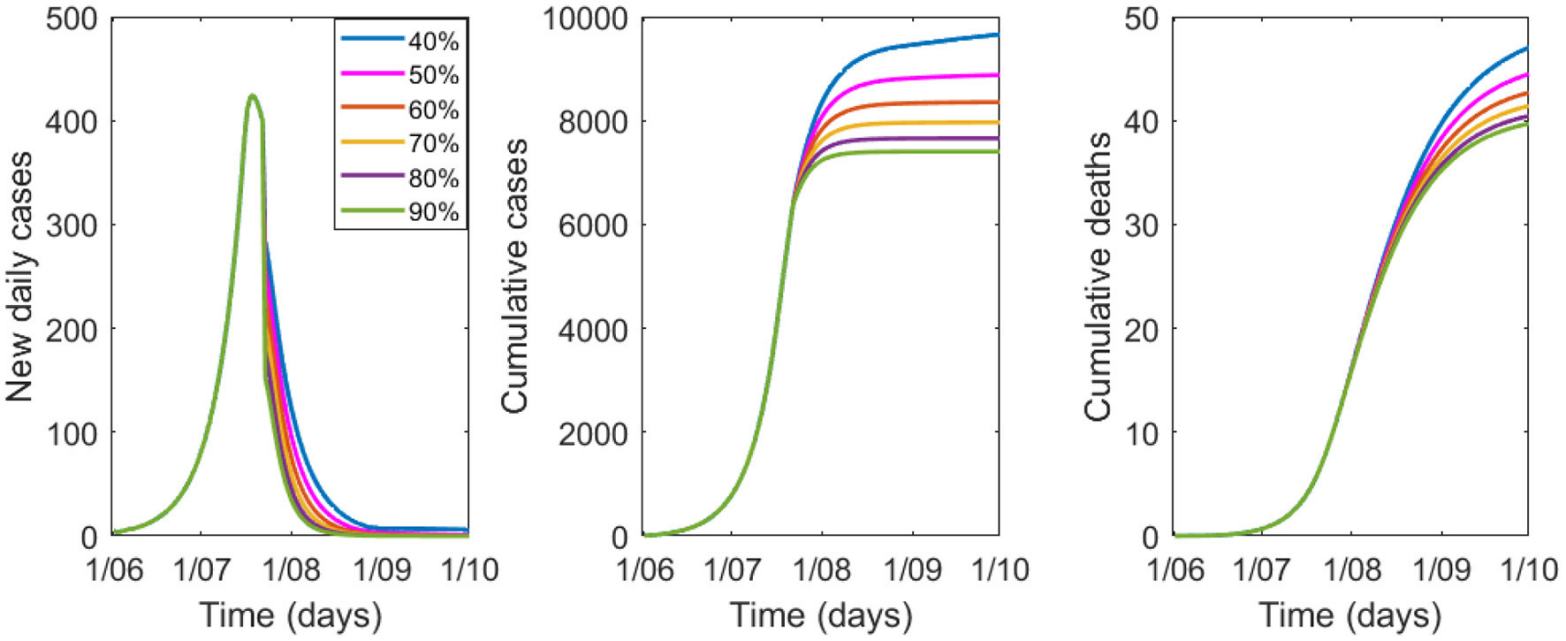
Variation in epidemic control for COVID-19 by percentage of people wearing masks from 40 to 90%, with a mask efficacy of 67%.

The sensitivity analysis on the percentage of people wearing masks is shown in Figure 3, Table 3. For good epidemic control, at least 40% of the population needs to wear good quality masks. If the proportion falls to 20% (see Table 3) the epidemic will result in 14,549 cases and 59 deaths by 1 October.

**Table 3:** Modeled cases and deaths by 1 October by percentage of the population wearing masks of 67% efficacy.

**People wearing masks (%)**	**Cases**	**Reduction in cases (%)**	**Deaths**	**Reduction in deaths (%)**
0	67,636	Base value	120	Base value
20	14,549	−78.5%	59	−50.8%
30	11,091	−83.6%	52	−56.7%
40	9,660	−85.7%	47	−60.8%
50	8,833	−86.9%	46	−61.7%
60	8,341	−87.7%	44	−63.3%
70	7,961	−88.2%	42	−65%
80	7,656	−88.7%	41	−65.8%
90	7,402	−89.1%	40	−66.7%

**Figure 4:**
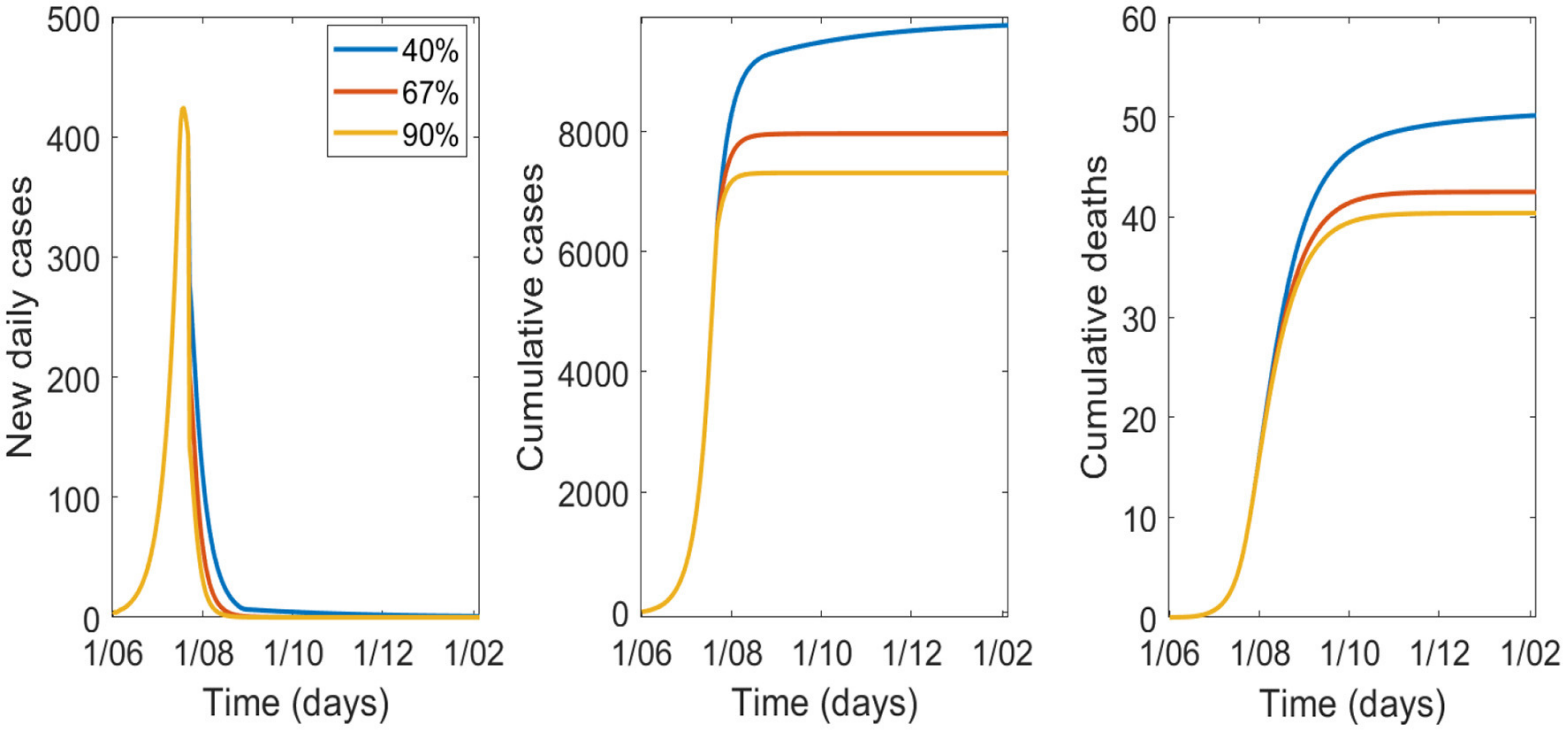
Variation in epidemic control for COVID-19 by varying effectiveness of masks from 40 to 90%, with 70% wearing a mask.

The sensitivity analysis for efficacy of masks is shown in Figure 4, Table 4 with 70% of the population wearing masks. Epidemic control is good with masks of effectiveness of 40% or greater.

**Table 4:** Modeled cases and deaths by 1 October by varying effectiveness of masks and 70% uptake of masks.

**Mask effectiveness %**	**Cases**	**Reduction in cases (%)**	**Deaths**	**Reduction in deaths (%)**
0	67,636	Base value	120	Base value
20	13,916	−79.4%	58	−51.7%
40	9,485	−86%	47	−60.8%
67	7,965	−88.2%	42	−65%
90	7,311	−89.2%	40	−66.7%

Finally, with an estimated 75% of the population using a mask during the second wave in Victoria (34), the model suggests that the effectiveness of masks was about 11%. With 75% of people wearing masks of 11% effectiveness the model shows 20,191 cases by 1 October, which is very close to the 20,183 cases officially reported by the same date (35).

## Discussion

Universal mask use likely contributed to epidemic control during the second wave in Victorian while a 6-week lockdown alone, without mask use, would have resulted in a much larger epidemic and a resurgence by September following the lifting of restrictions. We also show that the effect of masks increases with the increasing uptake and increased effectiveness of the masks. However, even modestly effective masks with uptake levels of 50% or greater, can substantially improve epidemic control. Masks should be seen as an important measure along with other NPIs during an epidemic of SARS-CoV-2 (1, 4, 36–38).

We assumed an effectiveness of 67% of masks in the base case scenario, which requires surgical masks or high-quality cloth masks to be used. The available data on cloth masks suggest a 12-layered mask is as effective as a surgical mask (4) but a 2-layered cotton mask may not be very effective (33). By fitting the model to observed cases and knowing the approximate uptake of masks at the time, we estimated that masks were 11% effective against SARS-COV-2 in Victoria, but that this still markedly reduced cases. The estimated real-world effectiveness of masks in Victoria was low, and likely reflects a wide range of poor-quality masks being used, including single-layered cloth face coverings of poor quality. Notice of the mask mandate was short, and over half of the population used cloth masks, likely home made (34). Cloth masks are the most accessible, feasible, and cost-effective option for the community, and research on improved design of cloth masks has been conducted during the pandemic (39, 40). Cloth masks made according to sound design principles, such as at least 3 layers, a water-resistant outer layer, good fit around the face may be as effective as a surgical mask (40). In addition, daily and adequate washing of cloth masks is required for effectiveness (41). Ensuring access to good quality cloth masks, avoiding low quality face coverings such as scarves or bandanas, and providing information on the best methods for making a high-quality mask will enhance protection.

We showed that early, pre-emptive use of facemasks could have prevented much of the epidemic in Victoria. This is challenging in a country where mask use is not a cultural norm, but early mask mandates are an important consideration for any area when experiencing community transmission.

At least 80% of all COVID-19 infections are mild, especially in younger people, which means that the majority of the burden of infection is at the milder end of the spectrum, as well as asymptomatic infection (12, 42). This also means that silent epidemic growth is possible before the scale of the epidemic is apparent. Many infectious people may not be apparent and may not themselves be aware of their infectiousness. The major benefit of mask use in the community is in preventing transmission that may not be easily identified, such as from mild, asymptomatic, or pre-symptomatic cases. Masks may in particular reduce the high risk of transmission in the 2 days prior to symptom onset and the first day of displaying symptoms (13, 18).

Masks may work by both protecting healthy people and preventing infected people from onward transmission. Seasonal coronaviruses are exhaled through normal breathing, and the emission of the virus on respiratory aerosols can be blocked completely by a surgical mask (43). In the US, the use of face masks by two infected hairdressers and by all 139 clients whom they serviced while infected, prevented transmission in the hair salon (44). However, one hairdresser did not wear a mask at home and infected household contacts. This provides some real-world evidence of the impact of universal mask use.

Our results are in line with other similar modeling studies. Two modeling studies, one using an SEIR model and a branching model (38), and the other using a next generation matrix model (45), tested mask effectiveness in the population against COVID-19. They found that wearing masks significantly reduced the spread, even when poor quality face masks were used, showing a very similar range of results, depending on slightly different parameter choices. Another SEIR model (46) has been used to support the same findings, where they showed that even low uptake of masks can have an impact on the epidemic curve. Our model structure is very similar to the one used by Eikenberry et al. (47).

The limitations of this study are the assumptions made for mask effectiveness. If the majority of people use very poor-quality cloth face coverings, the effectiveness would be much lower than the base case assumption of 67%—in fact, the estimation of effectiveness from the study was much lower than 67%. However, the sensitivity analysis of effectiveness estimates, ranging from poor quality cloth masks (20%) to N95 respirators (90%), encompasses all scenarios and shows that even low efficacy masks can have an impact on epidemic growth if there is sufficient uptake. We also assumed that the lockdown resulted in a 50% reduction in social contacts and mobility. If the reduction was greater, the impact of the lockdown would be greater. However, with people allowed to work and shop, schools being partially open and due to some parts of Victoria being exempt, we felt this was a reasonable assumption. A strength of our model is that we used viral shedding data to inform varying infectiousness over time, age-stratified disease parameters, as well as age-specific contact matrices. Some validation of the model was provided by the good model fit to the observed epidemic curve in Victoria.

Masks are an effective, cheap, low risk addition to other NPIs for the control of SARS-CoV-2 epidemic growth. No single NPI is adequate in controlling COVID-19, but used together, and used early, NPIs including masks can improve epidemic control (48). Pandemic planning could incorporate epidemic thresholds to trigger early mask mandates. Scaling up options for design and supply of good quality cloth masks can improve effectiveness and can be augmented by health promotion and education of community members.

## Data Availability Statement

The original contributions presented in the study are included in the article/supplementary material, further inquiries can be directed to the corresponding author/s.

## Author Contributions

CR: ideology, model parametrization, and writing and reviewing. VC: model builder and calibration and writing and reviewing.

## Conflict of Interest

The authors declare that the research was conducted in the absence of any commercial or financial relationships that could be construed as a potential conflict of interest.
